# Short- and long-term warming events on photosynthetic physiology, growth, and yields of field grown crops

**DOI:** 10.1042/BCJ20220433

**Published:** 2023-07-07

**Authors:** Carl J. Bernacchi, Ursula M. Ruiz-Vera, Matthew H. Siebers, Nicholas J. DeLucia, Donald R. Ort

**Affiliations:** 1 Global Change and Photosynthesis Research Unit, USDA-ARS, Urbana, IL, U.S.A.; 2 Department of Plant Biology, University of Illinois Urbana-Champaign, Urbana, IL, U.S.A.; 3 Carl R Woese Institute for Genomic Biology, University of Illinois Urbana-Champaign, Urbana, IL, U.S.A.; 4 Bayer CropScience LLC, Bayer Marana Greenhouse, Tucson, AZ 85743, U.S.A.

**Keywords:** crops, global warming, heat waves, photosynthesis

## Abstract

Global temperatures are rising from increasing concentrations of greenhouse gases in the atmosphere associated with anthropogenic activities. Global warming includes a warmer shift in mean temperatures as well as increases in the probability of extreme heating events, termed heat waves. Despite the ability of plants to cope with temporal variations in temperature, global warming is increasingly presenting challenges to agroecosystems. The impact of warming on crop species has direct consequences on food security, therefore understanding impacts and opportunities to adapt crops to global warming necessitates experimentation that allows for modification of growth environments to represent global warming scenarios. Published studies addressing crop responses to warming are extensive, however, in-field studies where growth temperature is manipulated to mimic global warming are limited. Here, we provide an overview of in-field heating techniques employed to understand crop responses to warmer growth environments. We then focus on key results associated with season-long warming, as expected with rising global mean temperatures, and with heat waves, as a consequence of increasing temperature variability and rising global mean temperatures. We then discuss the role of rising temperatures on atmospheric water vapor pressure deficit and potential implications for crop photosynthesis and productivity. Finally, we review strategies by which crop photosynthetic processes might be optimized to adapt crops to the increasing temperatures and frequencies of heat waves. Key findings from this review are that higher temperatures consistently reduce photosynthesis and yields of crops even as atmospheric carbon dioxide increases, yet potential strategies to minimize losses from high-temperature exist.

## Introduction

Emissions of greenhouse gases to the atmosphere are rising steadily associated with anthropogenic activities [1]. The impacts of rising greenhouse gases on the global climate system are far-reaching, with the most direct impact being an increase in global mean temperatures [2]. To date, anthropogenic greenhouse gas emissions have resulted in a ∼1°C increase in global temperatures although the increase is not uniform across the planet with terrestrial areas warming more than marine [2] . Concurrent with increasing mean temperatures is the increase in temperature variability [3]. While warmer mean temperature and greater temperature variance, independently, result in more extreme temperatures, combined warming, and increased temperature variability have a multiplicative impact on the frequency of extreme, high-temperature events. This is consistent with the trend toward more common and increasing intensity of heat waves [4].

Air temperatures fluctuate widely throughout multiple timescales ranging from within- to between days, over weeks, months, and seasons. As sessile organisms, plants experience this wide range of temperatures and as a result have developed myriad strategies to sense [5] and cope [6] with temperature changes and, to a limited extent, regulate their temperature [7,8]. Despite the adaptations that allow plants to thrive over a range of growth conditions, temperature is known to have a direct impact on all facets of biochemical and biophysical activities at the cellular, organ, and whole plant scale. Plant temperatures frequently deviate from air temperature as a consequence of absorption of radiation, transpiration, and a variety of other factors. Yet rising air temperatures and plant canopy temperatures are coupled through the energy budget which leads to direct increases in plant temperatures as air temperatures rise. While plants can thermally acclimate key metabolic processes when changes in growing conditions occur [9], these adaptations are likely intended for plant survival and not necessarily to maintain optimal physiological activity leading to maximum growth or fecundity. Projections suggest that plant temperatures that exceed 30°C result in potential yield losses for major crops [10], a threshold that is often exceeded [11], more so as the globe warms. Because of this, current and future projected global warming and increasing occurrences of extreme heat events are both likely to drive significant acclimation responses of plants and impact the physiological, morphological, growth, and reproductive success of plants growing in the terrestrial biosphere. Consequently, the reality of anthropogenically induced changes in atmospheric composition and warming portends future challenges to agriculture and global food security.

Photosynthesis accounts for the entry point for carbon into the terrestrial biosphere. As a result, any factors that impact photosynthetic rates are certain to influence the growth and yield of crops. Understanding how photosynthesis can be adapted to higher temperatures is pressing. Global temperatures have already exceeded optimal temperatures for photosynthesis in many locations and continued warming could lead to a 40% decrease in terrestrial biosphere productivity [12]. The general response of photosynthesis to short-term fluctuations in temperature has been characterized extensively for both C3 and C4 species [13] and the underlying parameterization necessary to predict photosynthesis over a wide range of temperatures has been successfully modeled [14–18]. While the importance of thermal acclimation of photosynthesis has been well established [9,19–21], actual adjustments in photosynthetic physiology that will occur with plants grown under higher temperatures over prolonged periods, consistent with global warming, are likely to alter growth and yield of crops under future climate conditions.

In this review, we outline the crop response of photosynthesis, growth, and yield to elevated temperature. Reproductive structures are known to be sensitive to elevated temperatures, however, in the context of this review, we will not focus on the mechanisms accounting for the temperature sensitivity of reproduction and instead recommend previous reviews on this topic (e.g. [22]). We will focus exclusively on field-based manipulative heating experiments, and, when appropriate, we will include interacting global change factors in addition to heating. The focus of the research presented in the manuscript is on canopy-scale heating of plants grown under field conditions as it is this scale that determines crop responses to the environment, that micrometeorological factors feedback on plant physiology and growth, and that global changes are likely to impact crop yields. Being a field-technique based review on major crops, we will focus on warming restricted to what are considered extreme temperature for major crop growing regions of the planet, where critical thresholds for heat stress are projected to occur at ∼30°C [10] and set an upper threshold of ca. 40°C to prevent extreme stress conditions that lead potentially to mortality. First, we will provide a summary of in-field heating technologies used to increase growth temperature for crops over long-term (prolonged heating) and short-term (heat wave) scenarios. This will be followed by a summary of responses observed for field crops grown under high-temperature environments. We will briefly outline confounding factors associated with in-field heating, particularly the impact on leaf-to-air vapor pressure deficit (VPD). Finally, we will discuss the potential for adapting crops to high-temperature growth environments.

## In-field heating technology to manipulate the growth environment for plants

A variety of heating methods have been used to increase canopy temperatures, but when studying global climate change, it is important to consider methods that most closely mimic future climate scenarios and what effects the treatment will have on data interpretation [23,24]. This section outlines the various methods used to increase the temperature of field-grown crops. Each technique listed will provide an overview of the technique, how it's been applied, and considerations/challenges associated with the technique.

Generally, methods that heat air can be categorized as either passive or active. The advantages and disadvantages of these methods have been reviewed and debated [25–29]. Passive techniques include field-installed greenhouses and passive open-top chambers. These structures trap long-wave radiation by partially or completely covering plots and increasing the boundary layer resistance which can more effectively trap heat. Despite a limited ability to control the amount of temperature change, it is a relatively low-cost, straightforward, and flexible method [30–32]. Because they are adaptable systems that require no power, passive structures are the preferred methods of temperature manipulation in remote ecosystems [27,33–35]. Furthermore, these passive systems can store humidity through the same mechanisms that trap heat, therefore potentially increasing the plant canopy temperature without increasing the VPD around the vegetation.

Active air heating techniques include open-top chambers, soil warming, and infrared (IR) heating elements that require powered infrastructure to modulate heating to warm temperatures above ambient. Open-top chambers typically use heating elements to warm air that is blown into the chamber to raise plot temperatures [36,37]. A benefit of open-top chambers includes the ability to alter many variables, e.g. raising CO_2_, tropospheric ozone, and/or temperature to study multiple global climate change factors with a single delivery system [38]. However, chamber structure and associated blowers have a significant effect on the microclimate beyond the intended treatments [39,40]. Resistance cables buried underground have been applied to studies focusing on understanding soil ecology impacts of global warming [41,42], however, they are not a practical method for increasing canopy temperatures.

**Figure 1.**
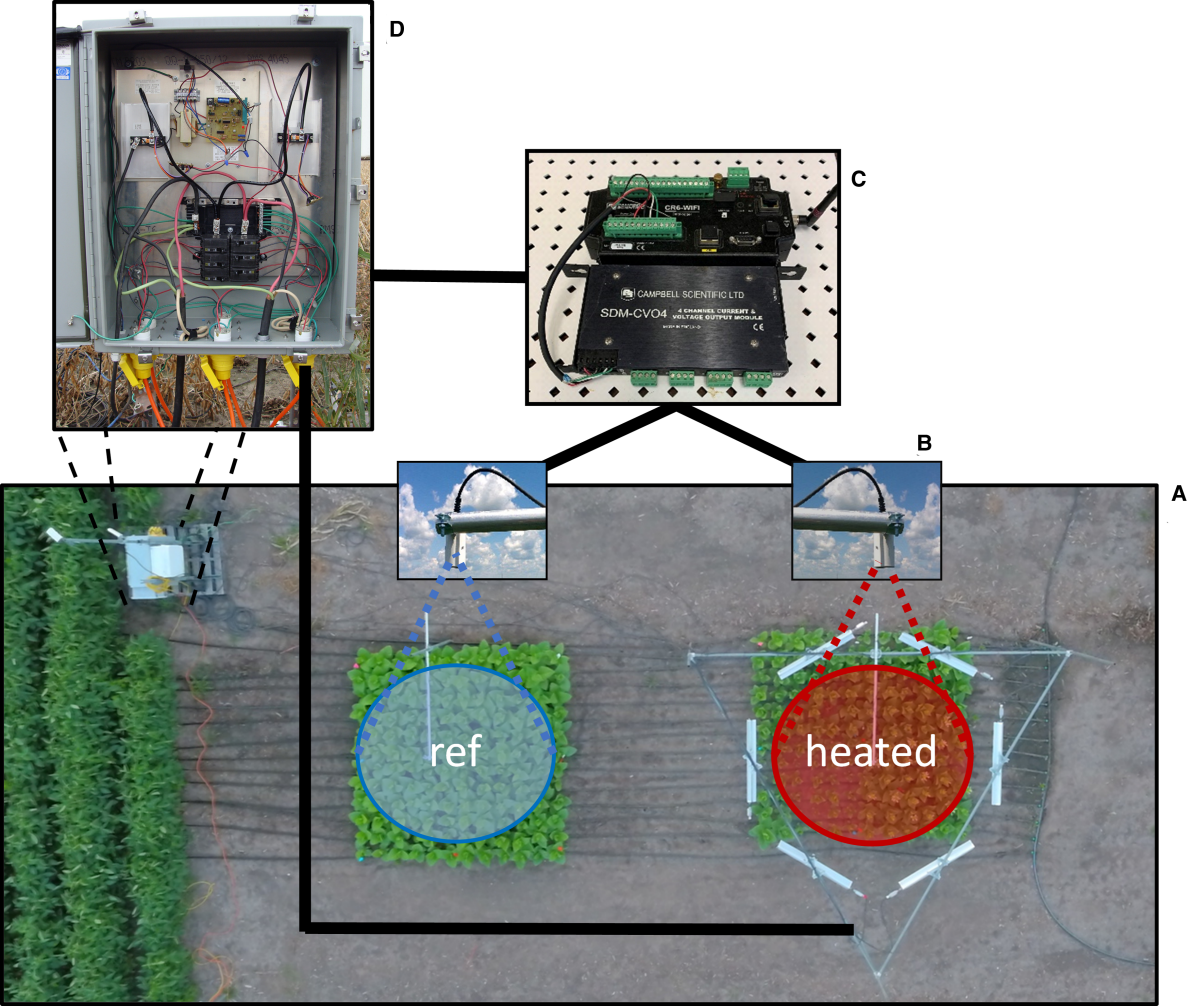
Schematic of an elevated temperature experiment [151]. (**A**) An aerial representation of a heated and reference plot representing one experimental block. The goal is to increase the heated plot temperature by a setpoint increase (°C) above the reference plot. The heated plot is surrounded by six heaters, each with four 1000 W heating elements, hexagonally arranged. An infrared thermometer (**B**) is mounted above both the heated and reference plot and wired into a datalogger (**C**, top) which is used to measure the surface temperature differences between the two plots. If the temperature difference between the plots differs from the target temperature setpoint increase for the heated plot, the datalogger uses a PID algorithm to adjust the programmable voltage output module (**C**, bottom) to a value between 0 and 10 V. The scalable output range (0–10 V) is wired into a heavy-duty industrial dimmer (**D**) to scale energy output (0 V = 0% output; 10 V = 100% output) to the heaters. Not shown are multiple circuit breakers and fused junctions, grounding, telecommunications, and enclosure lock-out power safety switches.

IR heating has become a widely used technique for increasing canopy temperatures in a range of ecosystems without the use of an enclosure [43–63], which is shown to heat somewhat consistently throughout the plant canopy profile (e.g. [64]). IR heating systems have undergone numerous advances since their inception for in-field heating purposes [47]. Some experiments set IR heater output to a constant level which causes small temperature differences during the day and large differences at night [59]. More commonly, IR heating arrays consist of several components (Figure 1), which work in unison to increase the temperature of a plant canopy to a setpoint above a reference plot that can be maintained or adjusted based on the research questions being investigated.

The key components needed to operate an IR heating array system are relatively straightforward (Figure 1). While there are a variety of techniques employed, the most common setup involves using IR radiometers to measure the temperature of a reference plot and a plot that is intended to be heated. These are non-contact sensors that are used to measure the temperature of a surface, at which they are pointed. The IR sensors are wired into a measurement and control peripheral that determines the reference plot temperature and is programmed to control IR heater output to maintain a user-defined heated treatment. IR heater control is usually accomplished by using an industrial dimmer with scaled voltage control interfaced with a voltage output module that is part of the measurement and control system. Specific details coupled with examples of each peripheral are provided in Figure 1 and elsewhere [49,53–55].

There are multiple challenges associated with construction and operation beyond the array components. First, high-powered utility service is required for system operation [54,56]. The heating arrays associated with the newest generation of heating systems range from 6 kW (e.g. [65]) to 24 kW (e.g. [55]) per array at maximum output, although actual output to maintain a setpoint will generally be lower than maximal output. Achieving maximum IR heater output requires nearby utility power and potentially dedicated transformers, which limits the potential locations where this technique can be applied. In rare cases when transformer availability was limited, a 37.5 kW diesel generator has been used by the authors as a stopgap at the Soybean Free Air CO_2_ Enrichment (SoyFACE; http://soyface.illinois.edu/) IR heating experiment. The second consideration is the cost of electricity consumption. Analysis of one plot resulted in an average seasonal mean electricity demand of 3.8 kW hours per day to achieve a 3.2°C temperature increase in the heated plot relative to the reference plot. However, the energy required to maintain heating is likely to vary substantially based on many factors including wind speed, target temperature increase, crop type, water availability, solar radiation, etc. Within the context of these costs is the need for replication. For example, a full-factorial elevated CO_2_ by warming experiment with four replicate blocks (*n* = 4) requires eight total arrays (e.g. [55]). Third, safety is a critical consideration when operating an IR heating experiment. Dangerously high electricity requirements coupled with extremely hot IR heating elements necessitate proper training, multiple fuse or circuit protection safety points, routine inspection of wires, professional contractors for key steps during setup, and personal protective equipment. Finally, as with most other in-field heating techniques, the use of IR heating arrays invariably increases the leaf-to-air VPD of the plants located within the heated treatment [28,49,66]. This results in the confounding influences of VPD and moisture feedbacks in addition to the intended warming [67], which, like all other active heating methods, challenges the assumption of heating as the main driver for physiological and growth responses [68,69].

## Crop photosynthetic responses to rising temperature

Photosynthesis is the primary pathway for carbon assimilation in plants, thus how photosynthesis responds to changes in temperature has a major impact on growth and development. The primary impact of rising temperatures on photosynthesis involves Ribulose-1,5-bisphosphate carboxylase-oxygenase (Rubisco), the primary enzyme to fix atmospheric CO_2_ in C3 plants. In addition to fixing CO_2_ to ribulose 1,5-bisphosphate (RuBP) to drive photosynthesis, C3 plants suffer from Rubisco oxygenation of RuBP, which initiates the energetically wasteful photorespiratory pathway [70]. Photorespiration has potential consequences for lower crop growth and yield. The carboxylation efficiency of Rubisco, defined as carbon fixation per photon of photosynthetically active radiation absorbed by the plants, is determined by many factors including the specificity of Rubisco for carboxylation vs. oxygenation (S_C/O_), Rubisco activation state, the concentrations of oxygen and carbon dioxide surrounding Rubisco, light intensity, and the rate of RuBP regeneration.

Decreased discrimination by Rubisco against oxygen, defined as a decline in S_C/O_, drive a decline in the rate of carboxylation (*V_c_*) relative to oxygenation (*V_o_*) as temperature increases [13,71–76]. For example, S_C/O_ in *Glycine max* (soybean) declines from ∼100 at 25°C to ∼90 at 30°C [77]. As a result, most C3 plants have a thermal optimum between 20 and 30 °C, with losses in photosynthetic potential above this optimum being driven by a lower *V*_c_/*V_o_*. Higher temperatures carry additional consequences related to declining concentration of CO_2_ relative to O_2_ in the chloroplast. As temperatures rise, the solubility of both O_2_ and CO_2_ declines, however, this decline is greater for CO_2_ than for O_2_. Additionally, mesophyll conductance in some species is shown to rise with temperature and remain relatively constant in others [16,78]. Even when *g_m_* increases it is insufficient to offset the impact of solubility and the decline in S_C/O_ with temperature leading to higher limitations photosynthetic carbon assimilation (e.g. [16]). In addition to the impact of higher temperatures on the supply of CO_2_ to Rubisco, the activation state of Rubisco is highly regulated by Rubisco activase [72,79]. Rubisco activase is shown to be highly temperature sensitive [74,80] and is shown to lower photosynthetic carboxylation efficiency through a decline in Rubisco activation state [81].

The C4 photosynthetic pathway effectively minimizes photorespiration by actively concentrating CO_2_ in the bundle sheath where Rubisco carboxylation occurs [82–87]. The exceptionally high concentration of CO_2_ achieved under most circumstances by C4 plants outcompetes oxygenation at biologically relevant temperatures, which leads to a higher temperature optimum for photosynthesis than C3 plants [13]. Furthermore, evidence suggests that Rubisco activase has a higher thermal stability in C4 plants than in most C3 crop species [88]. Despite these differences in photosynthetic responses of C3 and C4 species to temperature, photosynthetic acclimation to differing growth conditions can lead to plasticity in measured relative to theoretical responses [21,89]. This raises the importance of experimental approaches that manipulate growth temperatures for field-grown C3 and C4 crops both over the long-term (warming throughout the growing season) and short-term (high-intensity, shorter duration heat waves).

### Season-long elevated temperature

Season-long heating experiments provide valuable information to understand and predict the potential impacts of global warming on crop production. Multiple season-long experiments using IR heating arrays have been conducted on multiple crops species, including soybean (Glycine max; [55,90,91]), wheat (Triticum aestivum; [60,61,92,93]), rice (Oryza sativa; [63,94,95]), and maize (Zea mays; [91,96]).

**Figure 2.**
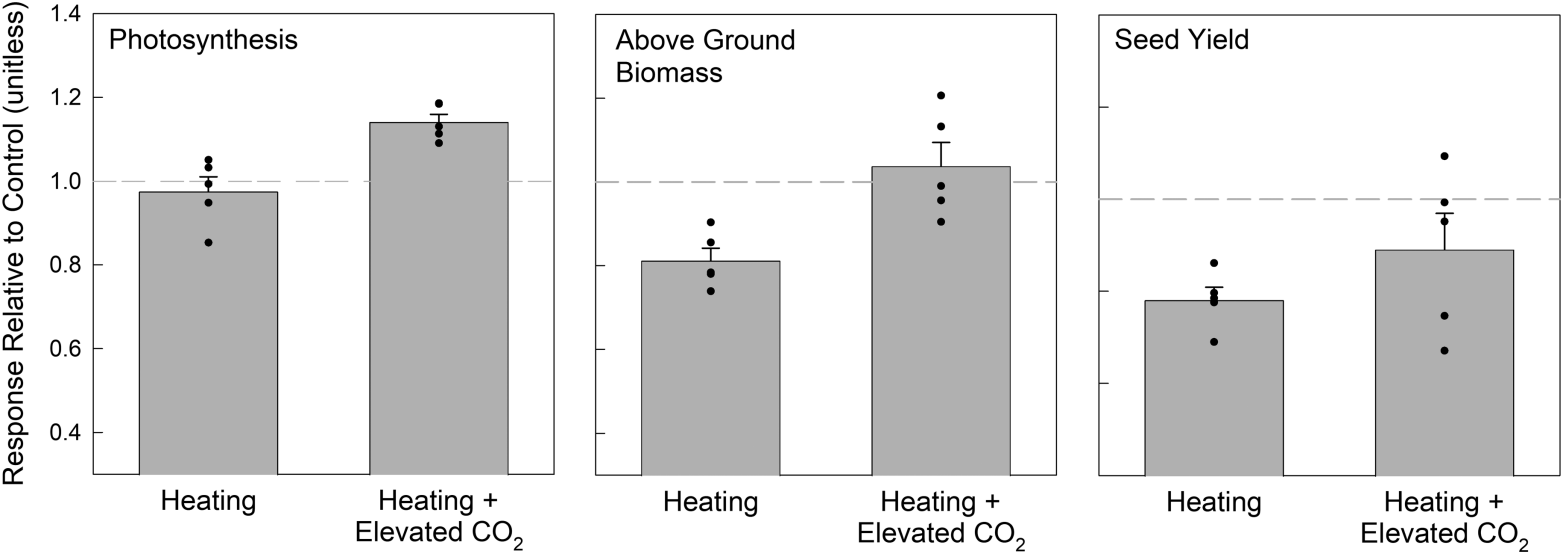
Summary of *in situ* photosynthesis, total above-ground biomass, and seed yield responses for wild-type soybean grown with season-long warming (heated) and season-long warming coupled with elevated CO_2_ (Heating + Elevated CO_2_) at the SoyFACE Research Facility in Urbana, IL. Photosynthesis data represents the mean *in situ* rates of photosynthesis collected midday over multiple measurement days during each season and averaged throughout the growing season. The above-ground biomass and seed yield data were collected at the end of each growing season. Target temperature increases were 3.5°C above background levels measured according to canopy temperature measurement. Full details for each measurement are described previously [55,97]. The bars represent the mean relative difference to the control (above 1 corresponds to an increase and below one a decrease in observed values relative to control), and error bars represent one standard error of the mean. Each symbol represents mean values for each growing season.

Soybean responses to elevated temperature at 3.5°C above background, with and without elevated CO_2_, have been investigated over five growing seasons [55,91,97]. Leaf photosynthetic rates varied in response to the heated treatments based on cultivar, back-ground meteorological conditions, and whether measured *in situ* midday [97] or integrated diurnally [55]. For most years, photosynthesis did not vary between control and elevated temperature treatments, except for one year when heating was applied to an already exceptionally warm year [55]. For this year the maximum canopy temperature in the heated treatment, set to 3.5°C above control plots, was the hottest of any year with daily maximum canopy temperatures ranging from 32.5°C to 41.5°C. This treatment saw reductions in photosynthesis by 15% [55]. These responses are supported by studies suggesting that the impact of warming on a region is dependent on the baseline conditions [98]. Despite the lack of differences among the treatments, observed maximum rates of electron transport (*J_max_*) were shown to be consistently lower for soybean grown in elevated temperatures [97,99]. Despite the variable responses associated with photosynthesis, above-ground biomass was consistently lower in elevated temperature relative to control for all years (Figure 2), which suggests that the leaf-level photosynthetic measurements may not be representative of whole-season responses and/or of canopy photosynthesis. Seed yield responses to elevated temperature were similar to those observed for above-ground biomass (Figure 2).

Soybean experiments have also evaluated the combined impact of elevated CO_2_ with warming [55,91,97,99]. Photosynthetic rates for the combined warming and elevated CO_2_ treatment were only slightly lower than observed for elevated CO_2_ alone (Figure 2) but rates for the combined treatment were much lower than the predicted synergistic effects based on modeling analysis [20,89]. This result is likely associated with both a reduction in stomatal conductance (gs; [55]) and a down-regulation of photosynthetic parameters [99] associated with both elevated CO_2_ and warmer temperatures. Above-ground biomass declined in the combined treatment to a greater extent than observed for photosynthesis, again suggesting that canopy-integrated photosynthetic responses may be greater than the responses observed at the leaf scale. Yield was reduced further than biomass in the combined treatment, likely due to the impacts of warming on photosynthesis, *g_s_*, and on reproductive development [90,91,96]. Furthermore, soybean grown in elevated CO_2_ shows consistently warmer canopy temperatures as a result of lower *g_s_* [100], thus increases in temperature associated with IR heating arrays and elevated CO_2_-induced declines in *g_s_* raise canopy temperatures further beyond the thermal optimum [55].

Wheat responses to growth in elevated temperature are shown to be highly variable relative to soybean. A sensitivity analysis of wheat yield responses to in-field heating of ∼1.5°C showed that winter wheat yield does not necessarily decrease with warmer temperatures in regions with cooler growing season temperatures or when the water supply is not limited [93]. Over a five-year experiment where temperatures were consistently lower than 25°C, wheat yield was shown to increase by ∼16% despite a temperature-induced shortening of the overall growing season. The yield increase was related to prolonged grain-filling developmental stages, relative to the non-heated plots, despite the overall shortened growing season [92,93]. In another experiment, plots of spring wheat were planted in staggered intervals over time from September to May (Fall to Spring) with supplemental warming of ∼1.3°C during the day and ∼2.7°C at night [61]. This experimental design allowed for variation in growth temperature associated with the staggered planting throughout the season coupled with the imposed heating conditions from the IR heaters. Results showed no yield impacts from supplemental heating when temperatures were near the optimum, and large yield increases with supplemental heating when background temperatures were below the optimum [61]. However, spring wheat yield was reduced in treatments where temperatures rose above the thermal optimum for wheat yield of ∼15°C [61].

Rice grown under in-field heating of ∼+1.5°C showed a reduced leaf area index, dry biomass, and yield [95]. Interestingly, these biomass and yield reductions were observed despite increases in the maximum rate of photosynthesis due to higher *V*_cmax_ and *J*_max_ in the heated treatment relative to control [95]. These results, like the ones observed in soybean, show that increases in photosynthesis are not always translated to increases in yield under a global warming scenario, and this can be related to other physiological processes, like grain development and grain growth. Consequently, warming accelerated rice development by reducing the days to heading and the days to get the maximum tiller number [94,101], which decreases spikelet density and consequently yield [101].

Despite being a C4 crop, maize grown under in-field heating resulted in reduced photosynthetic rates, again attributed to photosynthetic acclimation to higher temperatures [96]. No changes in *g_s_* and in intrinsic water use efficiency (iWUE) were observed for maize grown under high temperatures, relative to the control, which may be attributed to inherently lower *g_s_* associated with C4 species. Despite these photosynthetic responses, there was no observed difference in above-ground biomass for maize among the treatments, yet seed yield was lower in the heated treatments relative to the control, and this response was observed with and without elevated CO_2_ [96] . The lower yields in elevated temperature can potentially be attributed to lower photosynthetic rates, but higher temperatures led to more rapid progression through key developmental stages [91] and led to fewer kernels per cob [96] compared with the non-heated control, both of which are likely factors in the yield decline. The lower kernel count can be related to factors such as changes in silk receptivity, e.g. due to desiccation [102,103] and ear or kernel development, including kernel abortion [91,104–107], however, mechanistic understanding of these responses under in-field heating is still needed. While high temperature is likely to impact pollen viability [103,108], heated plots in these experiments are generally surrounded by non-heated vegetation, likely leading to an abundance of non-heat-treated and therefore fully viable pollen.

### Heat waves

Extreme heat events will become more intense and more frequent [4,109–112]. The definition of a heat wave is broad and because they are a major cause of weather-based mortality, definitions are often based on the amount of critical physiological stress posed to humans [113,114]. Studies on the effects of heat waves on plant growth and productivity are less common. Generally, there are two ways to study the effects of heat waves on plants. First one can use weather and historical yield data to model the effects of past extreme heat events on yield. Second, the heating technologies reviewed above can be used to impose heat waves in the field to help explore physiological breaking points.

Heat waves have significant effects on managed and unmanaged ecosystems [115]. A 2013 heat wave in southern China caused the largest crop yield anomaly in fifty years and reduced terrestrial carbon uptake in the region by ∼40–50% [116]. Similar heat waves have plagued other regions of the planet with a similar effect [117–119]. A useful framework has been provided for future heat wave research and outlines the role of compounding stresses and severe heat waves on crop failure and ecosystem die-off [120]. For crop loss in particular, it is clear that heat waves have the greatest impact on crop yield when they occur at a critical point of the growing season [121–123] and extreme temperatures play a major role in the projected yield reductions associated with climate change [124–126]. Given that temperature affects every biological process in the plant, it is important to simulate heat waves in the field as close to future projected conditions as possible during critical growth stages to identify processes that reduce yield.

Experimentally imposed heat waves in the field have large immediate effects on photosynthesis which tend to recover after the heat wave treatment. Heat waves that increased canopy temperatures 6°C above background temperatures for a three-day duration in soybean [123] and maize [122] consistently reduced photosynthetic rates during the heat waves, but photosynthesis recovered to control plot levels when the heatwave ended. From these same experiments, heat waves imposed during vegetative growth had no lasting impacts on yield, but heat waves imposed during sensitive reproductive stages in corn and soy caused significant decreases in yield [122,123]. An imposed three-day heat wave that increased the temperature to 38°C in wheat significantly reduced photosynthesis, but plants recovered completely within 30 days after the treatment ended [127]. In *Lens culinaris* (lentils), a three-day heat wave of 38–40 °C caused reductions in ΦPSII and electron transport rate, proxies for reduced photosynthesis [128]. The heat wave reduced lentil biomass by 6%, but yield was reduced by 30%, which suggests the sensitivity of reproductive structures to high-intensity heating.

There have been several FACE studies examining the interaction between elevated CO_2_ and heat waves on plant growth. In a study using lentils, elevated CO_2_ stimulated yield by 34%, but this yield increase disappeared when plants were grown in combined elevated CO_2_ and heat wave treatment [128]. Alternatively, the effects of elevated CO_2_ on moderate (+5°C) and extreme (+9°C) heat waves for soybean showed yield was maintained in the +5°C but not the +9°C treatment [129]. A low-intensity heat wave imposed on rice during reproductive development negatively impacted yields, and offset the potential benefit of elevated CO_2_ [63]. Ultimately, the impact of imposed heat waves on photosynthesis appears to be relatively limited to the duration of the heating event and has relatively minor impacts on total biomass, but appears to have a relatively consistent impact on yields when they are imposed during reproductive development. However, there are too few studies addressing the role of imposed heat waves on crops, necessitating more research to fully elucidate the underlying mechanisms and thresholds (duration and heating intensity) for responses.

A literature search did not yield any studies on a three-way interaction between elevated CO_2_, elevated seasonal temperature, and heat waves. From what we know about the acclimatory effects of elevated CO_2_ and season-long warming on C3 photosynthetic metabolism, heat waves may have an even greater effect on photosynthesis than has been measured. The photosynthetic acclimation to warming and elevated CO_2_ would lower photosynthetic rates at high temperatures and lower *g*_s_ [55,97,130], two things that are likely to happen during a heat wave. Alternatively, evidence exists that crops experiencing heat stress early in a growing season have a potential ‘priming’ effect that may provide protection later in the growing season [11]. Additional research on the effects of heat waves and the critical process that they affect are needed to understand, and potentially mitigate, scenarios that will lead to crop failure.

## In-field heating effects on crop water use efficiency through higher VPD

Atmospheric humidity is determined by the actual amount of water vapor in the atmosphere (actual water vapor pressure; *e_a_*) relative to the total amount of water vapor that the atmosphere can hold (saturation water vapor pressure; *e_s_*). The difference between *e_s_* and *e_a_* is termed VPD and is used as a metric for the atmospheric evaporative demand. Saturation vapor pressure increases exponentially as a function of temperature while *e_a_* is determined by water available to the atmosphere and is therefore highly variable across spatial and temporal scales. Rising global temperatures are resulting in higher *e_s_* [69,131–134] whereas the impact of global warming is having a lesser effect on *e_a_* [132]. Even in locations where *e_a_* is increasing, it is insufficient to offset the rise in *e_s_* leading to increases in VPD [135].

Disentangling the effects of higher temperatures and higher VPD on crops is essential given that differential increases in warming vs. atmospheric drying may elicit variable responses among crop types [136]. Plants respond to higher VPD by decreasing stomatal conductance [137]. The lower stomatal conductance conserves water loss associated with the higher evaporative demand but also can lower the CO_2_ flux into the leaves, lowers evaporative cooling, and, for C3 species and under some circumstances for C4 species, increase stomatal limitation to photosynthesis [138]. Given the tight linkage between temperature and VPD, separating physiological responses of plants to these variables, particularly under field conditions, is difficult. As a result, field-based understanding of how vegetation responds to heating vs. higher VPD is scarce in the literature. Despite major experimental challenges required to isolate physiological and mechanistic responses to both temperature and VPD independently, an improved understanding of the mechanisms of warming vs. drying would carry the potential for improved food security and, more accurate ecosystem modeling.

Rising VPD caused by global warming can have several impacts on plant growth and water use efficiency. High VPD can increase transpiration rates, which decreases water use efficiency and potentially can lead to increased water stress [68]. To avoid excess water loss and conserve water, *g_s_* generally decreases which may limit CO_2_ uptake for photosynthesis, also driving lower water use efficiency. Optimal VPD levels vary between plant species, but in general, a VPD range of 0.5–1.5 kPa is considered suitable for most plants. Overall, changes to VPD caused by global warming can have significant impacts on the growth, functionality, and water use efficiency of plants, which can have cascading effects on ecosystems and food production.

Experiments that address ecosystem responses to VPD suggest widespread reductions in productivity over a wide range of ecosystems based on a range of techniques and over many different scales but are generally limited to analysis of historic data or modeling output [134,139–141]. Rising VPD is shown to have significant impacts on crop physiology, primarily on *g_s_* and the subsequent impact of changes in *g_s_* on canopy and soil processes [68]. While much of this previous work shows the role of VPD on the growth and yield of crops, methods are primarily based on statistical analysis or models with a limited mechanistic representation of crop responses to VPD vs. temperature. Recently, a field-based humidifying system was developed and deployed for a northern European forest over whole growing seasons, showing significant impacts of decreasing VPD on tree species [142]. With the in-field IR heating arrays developed to test high-temperature impacts on crops, the addition of a similar in-field humidifying system could potentially lead to the ability to disentangle the impacts of warming temperature vs. rising VPD on crop growth, physiology, and yield. While more complicated than irrigating to offset increased evaporative demand, humidifying the air potentially removes the physiological responses to higher VPD associated with heating treatments. Carrying out an in-field humidification experiment poses some challenges much like the installation for the IR heating arrays. Adding humidity requires an infrastructure investment including a supply of water, a high-pressure misting system, a feedback control system, and electricity to pump the water. Furthermore, moist warm environments may increase the possibility of pathogens being present, so the systems require periodic inspections to treat for diseases before they impact crop functioning. Nevertheless, incorporating humidifying techniques into IR heating experiments can help understand the relative influences of heating plants without the consequent drying of the air around the plants.

## Adapting crops to high temperatures

Improving photosynthetic efficiency is essential to meet growing demands for agricultural products [143] yet, as outlined in this review, rising global temperatures are placing additional constraints on photosynthesis. The impact of warming temperature on photosynthesis defines a central target for adaptation as it is an important determinant of crop yield [144]. As the entry point for carbon into an ecosystem, photosynthesis is included in the list of likely targets for adapting crops to global warming [145,146]. In this section we review current strategies being investigated that can either minimize carbon losses or ideally increase carbon gains at higher temperatures.

Field research reviewed above points to the heat sensitivity of biochemical processes of carbon assimilation as the major limitation for both C3 and C4 photosynthesis. Overcoming the 10% reduction in S_c/o_ observed as plants are warmed from 25 to 30°C [77] would require a compensating increase in [CO_2_]/[O_2_] at the active site of Rubisco on the order of 10%. Although the predicted increase in atmospheric [CO_2_] to ∼550 ppm by the year 2050 will lower the impact of increased temperature on S_c/o_, it will not fully offset it [147]. The [CO_2_] in chloroplasts of C3 crops where carboxylation occurs will be affected by changes in stomatal and mesophyll conductances along with the larger reduction in the solubility of CO_2_ as compared with O_2_ with warmer temperatures. Increases in stomatal and mesophyll conductance are expected to increase net carboxylation, however, increased stomatal, but not mesophyll, conductance will also drive significantly more water loss. This suggests that increasing mesophyll conductance can be a potential target for crop adaption to higher temperatures as it will increase the CO_2_ in the chloroplast without increases in water loss. The temperature response of mesophyll conductance has been shown to increase or not change with increasing temperature depending upon species [16,78]. In the herbaceous species *Nicotiana tabacum*, *Gossypium hirsutum*, and *Glycine max* mesophyll conductance increased two- to threefold between 15 and 40°C, which is adaptive to the higher temperature. Increasing mesophyll conductance by exploiting natural variation or by engineering are thus potential strategies for adapting crops to warming.

The decrease in S_c/o_ that accompanies increased temperature results, at least in C3 plants, in a decrease in net photosynthesis due to increased photorespiration. Photorespiration is energetically very expensive, consuming as much as 30% of a plant's metabolic energy [147] to recycle the glycolate produced by Rubisco oxygenation. Less energetically expensive alternative transgenic pathways to recycle glycolate have been demonstrated in plants (e.g. [148–150]) for which theory predicts they should have their greatest benefit at high temperature where *V*_o_ and the consequent energetic costs are the highest. This prediction was confirmed with transgenic plants in which the native photorespiratory pathway had been bypassed, and glycolate flux was directed to a synthetic glycolate metabolism pathway installed in chloroplasts [151]. In canopy warming field experiments, it was shown that the substantial inhibition of growth by a season-long 5°C warming was largely prevented in plants containing the synthetic photorespiratory bypass. Another manipulation of photorespiration that may be adaptive to increasing growing season temperatures is the overexpression of the H-protein of glycine decarboxylase that has been shown to enhanced enhance net photosynthesis and growth of *Arabidopsis thaliana* [152] and *Nicotiana tabacum* [153]. While the mechanism by which the overexpression of this photorespiratory protein subunit stimulates photosynthesis is uncertain, there is again an expectation that it may have an increasing impact with rising Rubisco oxygenation rates with temperature.

The rate of regeneration of the CO_2_/O_2_ acceptor RuBP is also sensitive to temperature and variable with growth conditions and across species [154,155]. At current CO_2_ levels, light-saturated photosynthesis in C3 crop species operates at the intersection of Rubisco-limited and RuBP-limited photosynthesis, necessitating that photosynthesis will become increasingly limited by RuBP regeneration capacity as CO_2_ increases [156]. Additionally, RuBP regeneration limitation increases at higher temperatures [13], and RuBP-limited photosynthesis benefits from lower photorespiration at elevated CO_2_, implying that increases in the RuBP regeneration rate should raise the temperature optimum of photosynthesis at elevated CO_2_. Although there are multiple carbon reduction cycle enzymes along with the full suite of thylakoid membrane electron transport and ATP synthesis reactions involved in RuBP regeneration, modeling has consistently identified sedoheptulose-1,7-bisphosphatase, aldolase, and transketolase as exerting the greatest control [157,158]. There are numerous demonstrations confirming that the overexpression of sedoheptulose-1,7-bisphosphatase stimulates photosynthesis, implying a RuBP regeneration limitation [159], and protects against the inhibition of photosynthesis by moderate heat stress [160]. Moreover, transgenic expression of the cyanobacterial bifunctional fructose-1,6/sedoheptulose-1,7-bisphosphatase was shown to enhanced carbon assimilation and seed yield in soybean and resulted in significantly higher *V_c,max_* and *J_max_* under elevated CO_2_ and at elevated temperature protected against the seed yield loss that was seen in wild-type plants [97].

Rubisco activase (Rca), a nuclear-encoded chloroplast localized protein, regulates the proportion of Rubisco that is catalytically active by remodeling Rubisco to release inhibitory sugar phosphates from Rubisco active sites. The catalytic events that generate the compounds which inactivate Rubisco increase with temperature, causing progressive inhibition of Rubisco-limited photosynthesis during mild heat stress [161]. Paradoxically, Rca itself is sensitive to moderate heat stress in many species. Thermal stability of Rca was shown to be improved through gene shuffling to increase rates of photosynthesis and growth in genetically transformed Arabidopsis exposed to heat stress [162]. Nearly all plant species express two forms of Rca, the β form and the longer redox regulated α form. These isoforms come either from alternative splicing of the transcript of a single gene or from one or more copies of separate α and β isoform genes. For example, wheat has three Rca isoforms arising from one α and two β isoform genes. Heat induces the expression of one of the β genes suggesting that the heat stress response in wheat is in part mediated by this Rca β isoform [163]. *In vitro* studies confirmed that this β isoform is a heat-stable variant of Rca for wheat whereas the other β isoform is the most heat sensitive. The specific amino acids able to confer Rca thermostability to the wheat β isoform were identified [164]. It was further shown that substitution of a methionine residue with isoleucine in the heat-sensitive wheat Rca β isoform improved the thermal optimum without affecting the efficiency of Rubisco activation [165]. C4 grass species, such as *Sorghum bicolor* (sorghum), also have separate α and β genes, but in these grass species the Rca-α form is expressed only at temperatures above 42°C. Induction rate of Rca-α was shown to match the recovery rate of photosynthesis and Rubisco activation from the 42°C heat shock [88]. This association between Rca-α isoform expression and maintenance of Rubisco activation at high temperature suggests that Rca-α has a functional thermo-protective role in carbon fixation in C4 grasses by sustaining Rubisco activation at high temperature. These discoveries suggest that engineering Rca and/or manipulating its regulation could be possible strategies to improve Rubisco carboxylation efficiency in crop plants in a warming world but also reveal that due to the diversity in Rca regulation across species, the specific solutions for improving thermotolerance of photosynthesis via Rca engineering will differ across species.

## Conclusion

Whether the focus is on inherent intra- and inter-annual variability in growing temperature or the accelerating rate of global warming, understanding how crops respond to warming is critical to predict food security and to identify strategies to adapt crops to climate change. While there is still much more to understand about how warming can drive reductions in plant productivity, in-field heating technology is helping to provide some insights into crop responses and adaptation strategies to warming. Bridging field-based warming experiments with high-throughput phenotyping techniques, such as leaf spectral reflectance [166] and sun-induced chlorophyll fluorescence (SIF; [167]), are likely to accelerate understanding of crop photosynthetic physiological effects to warming over a range of temporal and spatial scales. These techniques are likely to create opportunities for future research on regional and global scale monitoring of crop responses to warming.

While our focus here is on the impact of increasing temperatures on photosynthesis, almost every biological process in a plant, from phenology to reproduction to water relations, is affected by high-temperature stress. Thus, while the warming climate does profoundly affect photosynthesis and photorespiration by various mechanisms, the diverse impacts of higher temperatures on other metabolic and physiological processes will unavoidably feedback on photosynthesis in ways that must be acknowledged and considered.

## Abbreviations

IR: Infrared
RuBP: ribulose 1,5-bisphosphate
VPD: vapor pressure deficit

## References

[1] Lamb, W.F., Wiedmann, T., Pongratz, J., Andrew, R., Crippa, M., Olivier, J.G.J. et al. (2021) A review of trends and drivers of greenhouse gas emissions by sector from 1990 to 2018. Environ. Res. Lett. 16, 073005 10.1088/1748-9326/abee4e

[2] IPCC. (2021) Climate Change 2021: The Physical Science Basis. Contribution of Working Group I to the Sixth Assessment Report of the Intergovernmental Panel on Climate Change, Cambridge University Press, Cambridge/New York, U.K./U.S.A.

[3] Hansen, J., Sato, M. and Ruedy, R. (2012) Perception of climate change. Proc. Natl Acad. Sci. U.S.A. 109, E2415–E2423 10.1073/pnas.1205276109 22869707 PMC3443154

[4] Perkins-Kirkpatrick, S.E. and Lewis, S.C. (2020) Increasing trends in regional heatwaves. Nat. Commun. 11, 3357 10.1038/s41467-020-16970-7 32620857 PMC7334217

[5] Ruelland, E. and Zachowski, A. (2010) How plants sense temperature. Environ. Exp. Bot. 69, 225–232 10.1016/j.envexpbot.2010.05.011

[6] Walbot, V. (2011) How plants cope with temperature stress. BMC Biol. 9, 79 10.1186/1741-7007-9-79 22093487 PMC3219733

[7] Michaletz, S.T., Weiser, M.D., Zhou, J., Kaspari, M., Helliker, B.R. and Enquist, B.J. (2015) Plant thermoregulation: energetics, trait–environment interactions, and carbon economics. Trends Ecol. Evol. 30, 714–724 10.1016/j.tree.2015.09.006 26476814

[8] Still, C.J., Rastogi, B., Page, G.F.M., Griffith, D.M., Sibley, A., Schulze, M. et al. (2021) Imaging canopy temperature: shedding (thermal) light on ecosystem processes. New Phytol. 230, 1746–1753 10.1111/nph.17321 33666251

[9] Yamori, W., Hikosaka, K. and Way, D.A. (2014) Temperature response of photosynthesis in C_3_, C_4_, and CAM plants: temperature acclimation and temperature adaptation. Photosynth. Res. 119, 101–117 10.1007/s11120-013-9874-6 23801171

[10] Schauberger, B., Archontoulis, S., Arneth, A., Balkovic, J., Ciais, P., Deryng, D. et al. (2017) Consistent negative response of US crops to high temperatures in observations and crop models. Nat. Commun. 8, 13931 10.1038/ncomms13931 28102202 PMC5253679

[11] Fu, P., Jaiswal, D., McGrath, J.M., Wang, S., Long, S.P. and Bernacchi, C.J. (2022) Drought imprints on crops can reduce yield loss: nature's insights for food security. Food Energy Security 11, e332 10.1002/fes3.332 35846892 PMC9285083

[12] Duffy, K.A., Schwalm, C.R., Arcus, V.L., Koch, G.W., Liang, L.L. and Schipper, L.A. (2021) How close are we to the temperature tipping point of the terrestrial biosphere? Sci. Adv. 7, eaay1052 10.1126/sciadv.aay1052 33523891 PMC7806211

[13] Sage, R.F. and Kubien, D.S. (2007) The temperature response of C_3_ and C_4_ photosynthesis. Plant Cell Environ. 30, 1086–1106 10.1111/j.1365-3040.2007.01682.x 17661749

[14] Bernacchi, C.J., Pimentel, C. and Long, S.P. (2003) *In vivo* temperature response functions of parameters required to model RuBP-limited photosynthesis. Plant Cell Environ. 26, 1419–1430 10.1046/j.0016-8025.2003.01050.x

[15] Bernacchi, C.J., Singsaas, E.L., Pimentel, C., Portis Jr, A.R. and Long, S.P. (2001) Improved temperature response functions for models of Rubisco-limited photosynthesis. Plant Cell Environ. 24, 253–259 10.1111/j.1365-3040.2001.00668.x

[16] Bernacchi, C.J., Portis, A.R., Nakano, H., von Caemmerer, S. and Long, S.P. (2002) Temperature response of mesophyll conductance. Implications for the determination of Rubisco enzyme kinetics and for limitations to photosynthesis *in vivo*. Plant Physiol. 130, 1992–1998 10.1104/pp.008250 12481082 PMC166710

[17] Bernacchi, C.J., Rosenthal, D.M., Pimentel, C., Long, S.P. and Farquhar, G.D. (2009) Modeling the temperature dependence of C_3_ photosynthesis. In Photosynthesis in Silico. Advances in Photosynthesis and Respiration (Laisk, A., Nedbal, L. and Govindjee, eds). pp. 231–246, Springer, Dordrecht

[18] Farquhar, G.D., von Caemmerer, S. and Berry, J.A. (1980) A biochemical model of photosynthetic CO_2_ assimilation in leaves of C_3_ species. Planta 149, 78–90 10.1007/BF00386231 24306196

[19] Dusenge, M.E., Duarte, A.G. and Way, D.A. (2019) Plant carbon metabolism and climate change: elevated CO_2_ and temperature impacts on photosynthesis, photorespiration and respiration. New Phytol. 221, 32–49 10.1111/nph.15283 29983005

[20] Long, S.P. (1991) Modification of the response of photosynthetic productivity to rising temperature by atmospheric CO_2_ concentrations: has its importance been underestimated? Plant Cell Environ. 14, 729–739 10.1111/j.1365-3040.1991.tb01439.x

[21] Way, D.A. and Yamori, W. (2014) Thermal acclimation of photosynthesis: on the importance of adjusting our definitions and accounting for thermal acclimation of respiration. Photosynth. Res. 119, 89–100 10.1007/s11120-013-9873-7 23812760

[22] Lohani, N., Singh, M.B. and Bhalla, P.L. (2019) High temperature susceptibility of sexual reproduction in crop plants. J. Exp. Bot. 71, 555–568 10.1093/jxb/erz426 31560053

[23] De Boeck, H.J., Vicca, S., Roy, J., Nijs, I., Milcu, A., Kreyling, J. et al. (2015) Global change experiments: challenges and opportunities. BioScience 65, 922–931 10.1093/biosci/biv099

[24] Ettinger, A., Chuine, I., Cook, B., Dukes, J., Ellison, A., Johnston, M. et al. (2019) How do climate change experiments alter plot-scale climate? Ecol. Lett. 22, 748–763 10.1111/ele.13223 30687988

[25] Asseng, S., Ewert, F., Martre, P., Rötter, R.P., Lobell, D.B., Cammarano, D. et al. (2015) Rising temperatures reduce global wheat production. Nat. Clim. Change 5, 143–147 10.1038/nclimate2470

[26] Amthor, J., Hanson, P., Norby, R. and Wullschleger, S. (2010) A comment on “Appropriate experimental ecosystem warming methods by ecosystem, objective, and practicality” by aronson and mcNulty. Agric. Forest Meteorol. 150, 497–498 10.1016/j.agrformet.2009.11.020

[27] Aronson, E.L. and McNulty, S.G. (2009) Appropriate experimental ecosystem warming methods by ecosystem, objective, and practicality. Agric. Forest Meteorol. 149, 1791–1799 10.1016/j.agrformet.2009.06.007

[28] Kimball, B.A. (2011) Comment on the comment by Amthor et al. on “Appropriate experimental ecosystem warming methods” by Aronson and McNulty. Agric. Forest Meteorol. 151, 420–424 10.1016/j.agrformet.2010.11.013

[29] Shaver, G.R., Canadell, J., Chapin, F.S., Gurevitch, J., Harte, J., Henry, G. et al. (2000) Global warming and terrestrial ecosystems: a conceptual framework for analysis. Bioscience 50, 871–882 10.1641/0006-3568(2000)050[0871:GWATEA]2.0.CO;2

[30] Frei, E.R., Schnell, L., Vitasse, Y., Wohlgemuth, T. and Moser, B. (2020) Assessing the effectiveness of *in-situ* active warming combined with open top chambers to study plant responses to climate change. Front. Plant Sci. 11, 539584 10.3389/fpls.2020.539584 33329621 PMC7714718

[31] Lu, X., O'Neill, C.M., Warner, S., Xiong, Q., Chen, X., Wells, R. et al. (2022) Winter warming post floral initiation delays flowering via bud dormancy activation and affects yield in a winter annual crop. Proc. Natl Acad. Sci. U.S.A. 119, e2204355119 10.1073/pnas.2204355119 36122201 PMC9522361

[32] Sadras, V., Moran, M. and Petrie, P. (2017) Resilience of grapevine yield in response to warming. Oeno One 51, 381–386 10.20870/oeno-one.2017.51.4.1913

[33] Bokhorst, S., Huiskes, A., Aerts, R., Convey, P., Cooper, E.J., Dalen, L. et al. (2013) Variable temperature effects of Open Top Chambers at polar and alpine sites explained by irradiance and snow depth. Glob. Chang. Biol. 19, 64–74 10.1111/gcb.12028 23504721

[34] Welshofer, K.B., Zarnetske, P.L., Lany, N.K. and Thompson, L.A. (2018) Open-top chambers for temperature manipulation in taller-stature plant communities. Methods Ecol. Evol. 9, 254–259 10.1111/2041-210X.12863

[35] Lewin, K.F., McMahon, A.M., Ely, K.S., Serbin, S.P. and Rogers, A. (2017) A zero-power warming chamber for investigating plant responses to rising temperature. Biogeosciences 14, 4071–4083 10.5194/bg-14-4071-2017

[36] Bader, M.Y., Moureau, E., Nikolić, N., Madena, T., Koehn, N. and Zotz, G. (2022) Simulating climate change in situ in a tropical rainforest understorey using active air warming and CO_2_ addition. Ecol. Evol. 12, e8406 10.1002/ece3.8406 35127002 PMC8796887

[37] Pelini, S.L., Bowles, F.P., Ellison, A.M., Gotelli, N.J., Sanders, N.J. and Dunn, R.R. (2011) Heating up the forest: open-top chamber warming manipulation of arthropod communities at Harvard and Duke Forests. Methods Ecol. Evol. 2, 534–540 10.1111/j.2041-210X.2011.00100.x

[38] Maynard, L.D., Moureau, E., Bader, M.Y., Salazar, D., Zotz, G. and Whitehead, S.R. (2022) Effects of climate change on plant resource allocation and herbivore interactions in a Neotropical rainforest shrub. Ecol. Evol. 12, e9198 10.1002/ece3.9198

[39] Long, S.P., Ainsworth, E.A., Leakey, A.D., Nosberger, J. and Ort, D.R. (2006) Food for thought: lower-than-expected crop yield stimulation with rising CO_2_ concentrations. Science 312, 1918–1921 10.1126/science.1114722 16809532

[40] McLeod, A. and Long, S. (1999) Free-air carbon dioxide enrichment (FACE) in global change research: a review. Adv. Ecol. Res. 28, 1–56 10.1016/S0065-2504(08)60028-8

[41] Melillo, J.M., Frey, S.D., DeAngelis, K.M., Werner, W.J., Bernard, M.J., Bowles, F.P. et al. (2017) Long-term pattern and magnitude of soil carbon feedback to the climate system in a warming world. Science 358, 101–105 10.1126/science.aan2874 28983050

[42] Sorensen, P.O., Finzi, A.C., Giasson, M.-A., Reinmann, A.B., Sanders-DeMott, R. and Templer, P.H. (2018) Winter soil freeze-thaw cycles lead to reductions in soil microbial biomass and activity not compensated for by soil warming. Soil Biol. Biochem. 116, 39–47 10.1016/j.soilbio.2017.09.026

[43] Bridgham, S.D., Pastor, J., Updegraff, K., Malterer, T.J., Johnson, K., Harth, C. et al. (1999) Ecosystem control over temperature and energy flux in northern peatlands. Ecol. Appl. 9, 1345–1358 10.1890/1051-0761(1999)009[1345:ECOTAE]2.0.CO;2

[44] Noormets, A., Chen, J., Bridgham, S.D., Weltzin, J.F., Pastor, J., Dewey, B. et al. (2004) The effects of infrared loading and water table on soil energy fluxes in Northern peatlands. Ecosystems 7, 573–582 10.1007/s10021-004-0013-2

[45] De Boeck, H.J., De Groote, T. and Nijs, I. (2012) Leaf temperatures in glasshouses and open-top chambers. New Phytol. 194, 1155–1164 10.1111/j.1469-8137.2012.04117.x 22448800

[46] Harte, J. and Shaw, R. (1995) Shifting dominance within a montane vegetation community: results of a climate-warming experiment. Science 267, 876–880 10.1126/science.267.5199.876 17813919

[47] Harte, J., Torn, M.S., Chang, F.-R., Feifarek, B., Kinzig, A.P., Shaw, R. et al. (1995) Global warming and soil microclimate: results from a meadow-warming experiment. Ecol. Appl. 5, 132–150 10.2307/1942058

[48] Kimball, B.A., White, J.W., Ottman, M.J., Wall, G.W., Bernacchi, C.J., Morgan, J. et al. (2015) Predicting canopy temperatures and infrared heater energy requirements for warming field plots. Agron. J. 107, 129–141 10.2134/agronj14.0109

[49] Kimball, B.A. (2005) Theory and performance of an infrared heater for ecosystem warming. Glob. Chang. Biol. 11, 2041–2056 10.1111/j.1365-2486.2005.1028.x

[50] Kimball, B.A. (2015) Using canopy resistance for infrared heater control when warming open-field plots. Agron. J. 107, 1105–1112 10.2134/agronj14.0418

[51] Kimball, B.A., Alonso-Rodríguez, A.M., Cavaleri, M.A., Reed, S.C., González, G. and Wood, T.E. (2018) Infrared heater system for warming tropical forest understory plants and soils. Ecol. Evol. 8, 1932–1944 10.1002/ece3.3780 29468013 PMC5817131

[52] Kimball, B.A. and Conley, M.M. (2009) Infrared heater arrays for warming field plots scaled up to 5-m diameter. Agric. Forest Meteorol. 149, 721–724 10.1016/j.agrformet.2008.09.015

[53] Kimball, B.A., Conley, M.M. and Lewin, K.F. (2012) Performance and energy costs associated with scaling infrared heater arrays for warming field plots from 1 to 100 m. Theor. Appl. Climatol. 108, 247–265 10.1007/s00704-011-0518-5

[54] Kimball, B.A., Conley, M.M., Wang, S., Lin, X., Luo, C., Morgan, J. et al. (2008) Infrared heater arrays for warming ecosystem field plots. Glob. Chang. Biol. 14, 309–320 10.1111/j.1365-2486.2007.01486.x

[55] Ruiz-Vera, U.M., Siebers, M., Gray, S.B., Drag, D.W., Rosenthal, D.M., Kimball, B.A. et al. (2013) Global warming can negate the expected CO_2_ stimulation in photosynthesis and productivity for soybean grown in the midwestern United States. Plant Physiol. 162, 410–423 10.1104/pp.112.211938 23512883 PMC3641220

[56] Nijs, I., Kockelbergh, F., Teughels, H., Blum, H., Hendrey, G. and Impens, I. (1996) Free air temperature increase (FATI): a new tool to study global warming effects on plants in the field. Plant Cell Environ. 19, 495–502 10.1111/j.1365-3040.1996.tb00343.x

[57] Nijs, I., Teughels, H., Blum, H., Hendrey, G. and Impens, I. (1996) Simulation of climate change with infrared heaters reduces the productivity of *Lolium perenne* L. in summer. Environ. Exp. Bot. 36, 271–280 10.1016/0098-8472(96)01021-0

[58] Shaw, M.R., Zavaleta, E.S., Chiariello, N.R., Cleland, E.E., Mooney, H.A. and Field, C.B. (2002) Grassland responses to global environmental changes suppressed by elevated CO_2_. Science 298, 1987–1990 10.1126/science.1075312 12471257

[59] Wan, S., Luo, Y. and Wallace, L.L. (2002) Changes in microclimate induced by experimental warming and clipping in tallgrass prairie. Glob. Chang. Biol. 8, 754–768 10.1046/j.1365-2486.2002.00510.x

[60] Wall, G.W., Kimball, B.A., White, J.W. and Ottman, M.J. (2011) Gas exchange and water relations of spring wheat under full-season infrared warming. Glob. Chang. Biol. 17, 2113–2133 10.1111/j.1365-2486.2011.02399.x

[61] Ottman, M., Kimball, B., White, J. and Wall, G. (2012) Wheat growth response to increased temperature from varied planting dates and supplemental infrared heating. Agron. J. 104, 7–16 10.2134/agronj2011.0212

[62] Webber, H., Martre, P., Asseng, S., Kimball, B., White, J., Ottman, M. et al. (2017) Canopy temperature for simulation of heat stress in irrigated wheat in a semi-arid environment: a multi-model comparison. Field Crops Res. 202, 21–35 10.1016/j.fcr.2015.10.009

[63] Zhang, G., Ujiie, K., Yoshimoto, M., Sakai, H., Tokida, T., Usui, Y. et al. (2022) Daytime warming during early grain filling offsets the CO_2_ fertilization effect in rice. Environ. Res. Lett. 17, 114051 10.1088/1748-9326/aca038

[64] Siebers, M. (2014) Impacts of Heat Waves on Food Quantity and Quality of Soybean/Corn in the Midwest at Ambient and Elevated [CO_2_], University of Illinois at Urbana-Champaign, Urbana

[65] Grant, R., Kimball, B., Conley, M., White, J., Wall, G. and Ottman, M. (2011) Controlled warming effects on wheat growth and yield: field measurements and modeling. Agron. J. 103, 1742–1754 10.2134/agronj2011.0158

[66] Norby, R., Edwards, N., Riggs, J., Abner, C., Wullschleger, S. and Gunderson, C. (1997) Temperature-controlled open-top chambers for global change research. Glob. Chang. Biol. 3, 259–267 10.1046/j.1365-2486.1997.00072.x

[67] De Boeck, H.J., Kimball, B.A., Miglietta, F. and Nijs, I. (2012) Quantification of excess water loss in plant canopies warmed with infrared heating. Glob. Chang. Biol. 18, 2860–2868 10.1111/j.1365-2486.2012.02734.x 24501063

[68] Grossiord, C., Buckley, T.N., Cernusak, L.A., Novick, K.A., Poulter, B., Siegwolf, R.T.W. et al. (2020) Plant responses to rising vapor pressure deficit. New Phytol. 226, 1550–1566 10.1111/nph.16485 32064613

[69] Novick, K.A., Ficklin, D.L., Stoy, P.C., Williams, C.A., Bohrer, G., Oishi, A.C. et al. (2016) The increasing importance of atmospheric demand for ecosystem water and carbon fluxes. Nat. Clim. Chang. 6, 1023–1027 10.1038/nclimate3114

[70] Ogren, W.L. (1984) Photorespiration: pathways, regulation, and modification. Annu. Rev. Plant Physiol. 35, 415–442 10.1146/annurev.pp.35.060184.002215

[71] Badger, M. and Andrews, T. (1974) Effects of CO_2_, O_2_ and temperature on a high-affinity form of ribulose diphosphate carboxylase-oxygenase from spinach. Biochem. Biophys. Res. Commun. 60, 204–210 10.1016/0006-291x(74)90192-2 4418741

[72] Crafts-Brandner, S.J., van de Loo, F.J. and Salvucci, M.E. (1997) The two forms of ribulose-1, 5-bisphosphate carboxylase/oxygenase Activase differ in sensitivity to elevated temperature. Plant Physiol. 114, 439–444 10.1104/pp.114.2.439 12223718 PMC158323

[73] Salvucci, M.E. and Crafts-Brandner, S.J. (2004) Inhibition of photosynthesis by heat stress: the activation state of Rubisco as a limiting factor in photosynthesis. Physiol. Plant. 120, 179–186 10.1111/j.0031-9317.2004.0173.x 15032851

[74] Perdomo, J.A., Capó-Bauçà, S., Carmo-Silva, E. and Galmés, J. (2017) Rubisco and rubisco Activase play an important role in the biochemical limitations of photosynthesis in rice, wheat, and maize under high temperature and water deficit. Front. Plant Sci. 8, 490 10.3389/fpls.2017.00490 28450871 PMC5390490

[75] Perdomo, J.A., Carmo-Silva, E., Hermida-Carrera, C., Flexas, J. and Galmés, J. (2016) Acclimation of biochemical and diffusive components of photosynthesis in rice, wheat, and maize to heat and water deficit: implications for modeling photosynthesis. Front. Plant Sci. 7, 1719 10.3389/fpls.2016.01719 27920782 PMC5118457

[76] Rashid, M.A., Andersen, M.N., Wollenweber, B., Kørup, K., Zhang, X. and Olesen, J.E. (2018) Impact of heat-wave at high and low VPD on photosynthetic components of wheat and their recovery. Environ. Exp. Bot. 147, 138–146 10.1016/j.envexpbot.2017.12.009

[77] Orr, D.J., Alcântara, A., Kapralov, M.V., Andralojc, P.J., Carmo-Silva, E. and Parry, M.A. (2016) Surveying Rubisco diversity and temperature response to improve crop photosynthetic efficiency. Plant Physiol. 172, 707–717 10.1104/pp.16.00750 27342312 PMC5047088

[78] von Caemmerer, S. and Evans, J.R. (2015) Temperature responses of mesophyll conductance differ greatly between species. Plant Cell Environ. 38, 629–637 10.1111/pce.12449 25224884

[79] Portis, Jr, A.R., Li, C., Wang, D. and and Salvucci, M.E. (2008) Regulation of Rubisco activase and its interaction with Rubisco. J. Exp. Bot. 59, 1597–1604 10.1093/jxb/erm240 18048372

[80] Salvucci, M.E., Osteryoung, K.W., Crafts-Brandner, S.J. and Vierling, E. (2001) Exceptional sensitivity of Rubisco activase to thermal denaturation *in vitro* and *in vivo*. Plant Physiol. 127, 1053–1064 10.1104/pp.010357 11706186 PMC129275

[81] Sage, R.F., Way, D.A. and Kubien, D.S. (2008) Rubisco, Rubisco activase, and global climate change. J. Exp. Bot. 59, 1581–1595 10.1093/jxb/ern053 18436544

[82] Furbank, R.T. and Hatch, M.D. (1987) Mechanism of C_4_ photosynthesis: the size and composition of the inorganic carbon pool in bundle sheath cells. Plant Physiol. 85, 958–964 10.1104/pp.85.4.958 16665838 PMC1054376

[83] Hatch, M.D. (1992) C_4_ photosynthesis: an unlikely process full of surprises. Plant Cell Physiol. 33, 333–342 10.1093/oxfordjournals.pcp.a078260

[84] Hatch, M.D. (2005) C_4_ photosynthesis: discovery and resolution. In Discoveries in Photosynthesis (Govindjee, Beatty, J.T., Gest, H., Allen, J.F.), pp. 875–880, Springer, Dordrecht

[85] He, D. and Edwards, G.E. (1996) Estimation of diffusive resistance of bundle sheath cells to CO_2_ from modeling of C_4_ photosynthesis. Photosynth. Res. 49, 195–208 10.1007/BF00034781 24271698

[86] Kiirats, O., Lea, P.J., Franceschi, V.R. and Edwards, G.E. (2002) Bundle sheath diffusive resistance to CO_2_ and effectiveness of C4 photosynthesis and refixation of photorespired CO_2_ in a C4 cycle mutant and wild-type *Amaranthus edulis*. Plant Physiol. 130, 964–976 10.1104/pp.008201 12376660 PMC166622

[87] von Caemmerer, S. and Furbank, R.T. (2003) The C4 pathway: an efficient CO_2_ pump. Photosynth. Res. 77, 191–207 10.1023/A:1025830019591 16228376

[88] Kim, S.Y., Slattery, R.A. and Ort, D.R. (2021) A role for differential Rubisco activase isoform expression in C4 bioenergy grasses at high temperature. Glob. Chang. Biol. Bioenergy 13, 211–223 10.1111/gcbb.12768

[89] Bagley, J.E., Miller, J. and Bernacchi, C.J. (2015) Biophysical impacts of climate-smart agriculture in the Midwest United States. Plant Cell Environ. 38, 1913–1930 10.1111/pce.12485 25393245

[90] Leisner, C.P., Yendrek, C.R. and Ainsworth, E.A. (2017) Physiological and transcriptomic responses in the seed coat of field-grown soybean (Glycine max L. Merr.) to abiotic stress. BMC Plant Biol. 17, 242 10.1186/s12870-017-1188-y 29233093 PMC5727933

[91] Ruiz-Vera, U.M., Siebers, M.H., Jaiswal, D., Ort, D.R. and Bernacchi, C.J. (2018) Canopy warming accelerates development in soybean and maize, offsetting the delay in soybean reproductive development by elevated CO_2_ concentrations. Plant Cell Environ. 41, 2806–2820 10.1111/pce.13410 30055106

[92] Tian, Y., Chen, J., Chen, C., Deng, A., Song, Z., Zheng, C. et al. (2012) Warming impacts on winter wheat phenophase and grain yield under field conditions in Yangtze Delta Plain, China. Field Crops Res. 134, 193–199 10.1016/j.fcr.2012.05.013

[93] Zhao, C., Piao, S., Huang, Y., Wang, X., Ciais, P., Huang, M. et al. (2016) Field warming experiments shed light on the wheat yield response to temperature in China. Nat. Commun. 7, 13530 10.1038/ncomms13530 27853151 PMC5118553

[94] Usui, Y., Sakai, H., Tokida, T., Nakamura, H., Nakagawa, H. and Hasegawa, T. (2016) Rice grain yield and quality responses to free-air CO_2_ enrichment combined with soil and water warming. Glob. Chang. Biol. 22, 1256–1270 10.1111/gcb.13128 26463894

[95] Wang, W., Cai, C., He, J., Gu, J., Zhu, G., Zhang, W. et al. (2020) Yield, dry matter distribution and photosynthetic characteristics of rice under elevated CO_2_ and increased temperature conditions. Field Crops Res. 248, 107605 10.1016/j.fcr.2019.107605

[96] Ruiz-Vera, U.M., Siebers, M.H., Drag, D.W., Ort, D.R. and Bernacchi, C.J. (2015) Canopy warming caused photosynthetic acclimation and reduced seed yield in maize grown at ambient and elevated [CO_2_]. Glob. Chang. Biol. 21, 4237–4249 10.1111/gcb.13013 26119211

[97] Köhler, I.H., Ruiz-Vera, U.M., VanLoocke, A., Thomey, M.L., Clemente, T., Long, S.P. et al. (2017) Expression of cyanobacterial FBP/SBPase in soybean prevents yield depression under future climate conditions. J. Exp. Bot. 68, 715–726 10.1093/jxb/erw435 28204603 PMC5441901

[98] Hatfield, J.L., Boote, K.J., Kimball, B.A., Ziska, L., Izaurralde, R.C., Ort, D. et al. (2011) Climate impacts on agriculture: implications for crop production. Agron. J. 103, 351–370 10.2134/agronj2010.0303

[99] Rosenthal, D.M., Ruiz-Vera, U.M., Siebers, M.H., Gray, S.B., Bernacchi, C.J. and Ort, D.R. (2014) Biochemical acclimation, stomatal limitation and precipitation patterns underlie decreases in photosynthetic stimulation of soybean (*Glycine max*) at elevated [CO_2_] and temperatures under fully open air field conditions. Plant Sci. 226, 136–146 10.1016/j.plantsci.2014.06.013 25113459

[100] Bernacchi, C.J., Kimball, B.A., Quarles, D.R., Long, S.P. and Ort, D.R. (2007) Decreases in stomatal conductance of soybean under open-air elevation of [CO_2_] are closely coupled with decreases in ecosystem evapotranspiration. Plant Physiol. 143, 134–144 10.1104/pp.106.089557 17114275 PMC1761983

[101] Wang, W., Cai, C., Lam, S.K., Liu, G. and Zhu, J. (2018) Elevated CO_2_ cannot compensate for japonica grain yield losses under increasing air temperature because of the decrease in spikelet density. Eur. J. Agron. 99, 21–29 10.1016/j.eja.2018.06.005

[102] Heslop-Harrison, J. (1979) An interpretation of the hydrodynamics of pollen. Am. J. Bot. 66, 737–743 10.1002/j.1537-2197.1979.tb06277.x

[103] Schoper, J.B., Lambert, R.J., Vasilas, B.L. and Westgate, M.E. (1987) Plant factors controlling seed set in maize: the influence of silk, pollen, and ear-leaf water status and tassel heat treatment at pollination. Plant Physiol. 83, 121–125 10.1104/pp.83.1.121 16665186 PMC1056309

[104] Setter, T.L., Flannigan, B.A. and Melkonian, J. (2001) Loss of kernel set due to water deficit and shade in maize: carbohydrate supplies, abscisic acid, and cytokinins. Crop Sci. 41, 1530–1540 10.2135/cropsci2001.4151530x

[105] Zinselmeier, C., Jeong, B.-R. and Boyer, J.S. (1999) Starch and the control of kernel number in maize at low water potentials. Plant Physiol. 121, 25–36 10.1104/pp.121.1.25 10482657 PMC59374

[106] Westgate, M.E. and Boyer, J.S. (1986) Reproduction at low and pollen water potentials in maize. Crop Sci. 26, 951–956 10.2135/cropsci1986.0011183X002600050023x

[107] Westgate, M.E. and Hatfield, J.L. (2011) Genetic adjustment to changing climates: maize. In Crop Adaptation to Climate Change (Yadav, S.S., Redden, R.J., Hatfield, J.L., Lotze-Campen, H. and Hall, A.E., eds), pp. 314–325, Wiley-Blackwell, Chichester

[108] Fonseca, A.E. and Westgate, M.E. (2005) Relationship between desiccation and viability of maize pollen. Field Crops Res. 94, 114–125 10.1016/j.fcr.2004.12.001

[109] Bathiany, S., Dakos, V., Scheffer, M. and Lenton, T. (2018) Climate models predict increasing temperature variability in poor countries. Sci. Adv. 4, eaar5809 10.1126/sciadv.aar5809 29732409 PMC5931768

[110] Holmes, C.R., Woollings, T., Hawkins, E. and De Vries, H. (2016) Robust future changes in temperature variability under greenhouse gas forcing and the relationship with thermal advection. J. Clim. 29, 2221–2236 10.1175/JCLI-D-14-00735.1

[111] Meehl, G.A. and Tebaldi, C. (2004) More intense, more frequent, and longer lasting heat waves in the 21st century. Science 305, 994–997 10.1126/science.1098704 15310900

[112] Vautard, R., van Aalst, M., Boucher, O., Drouin, A., Haustein, K., Kreienkamp, F. et al. (2020) Human contribution to the record-breaking June and July 2019 heatwaves in Western Europe. Environ. Res. Lett. 15, 094077 10.1088/1748-9326/aba3d4

[113] Robinson, P.J. (2001) On the definition of a heat wave. J. Appl. Meteorol. Climatol. 40, 762–775 10.1175/1520-0450(2001)040<0762:OTDOAH>2.0.CO;2

[114] Xu, Z., FitzGerald, G., Guo, Y., Jalaludin, B. and Tong, S. (2016) Impact of heatwave on mortality under different heatwave definitions: a systematic review and meta-analysis. Environ. Int. 89, 193–203 10.1016/j.envint.2016.02.007 26878285

[115] Brás, T.A., Seixas, J., Carvalhais, N. and Jägermeyr, J. (2021) Severity of drought and heatwave crop losses tripled over the last five decades in Europe. Environ. Res. Lett. 16, 065012 10.1088/1748-9326/abf004

[116] Yuan, W., Cai, W., Chen, Y., Liu, S., Dong, W., Zhang, H. et al. (2016) Severe summer heatwave and drought strongly reduced carbon uptake in Southern China. Sci. Rep. 6, 18813 10.1038/srep18813 26739761 PMC4703972

[117] Bastos, A., Ciais, P., Friedlingstein, P., Sitch, S., Pongratz, J., Fan, L. et al. (2020) Direct and seasonal legacy effects of the 2018 heat wave and drought on European ecosystem productivity. Sci. Adv. 6, eaba2724 10.1126/sciadv.aba2724 32577519 PMC7286671

[118] Ciais, P., Reichstein, M., Viovy, N., Granier, A., Ogée, J., Allard, V. et al. (2005) Europe-wide reduction in primary productivity caused by the heat and drought in 2003. Nature 437, 529–533 10.1038/nature03972 16177786

[119] Reichstein, M., Bahn, M., Ciais, P., Frank, D., Mahecha, M.D., Seneviratne, S.I. et al. (2013) Climate extremes and the carbon cycle. Nature 500, 287–295 10.1038/nature12350 23955228

[120] Breshears, D.D., Fontaine, J.B., Ruthrof, K.X., Field, J.P., Feng, X., Burger, J.R. et al. (2021) Underappreciated plant vulnerabilities to heat waves. New Phytol. 231, 32–39 10.1111/nph.17348 33728638

[121] Ortiz-Bobea, A., Wang, H., Carrillo, C.M. and Ault, T.R. (2019) Unpacking the climatic drivers of US agricultural yields. Environ. Res. Lett. 14, 064003 10.1088/1748-9326/ab1e75

[122] Siebers, M.H., Slattery, R.A., Yendrek, C.R., Locke, A.M., Drag, D., Ainsworth, E.A. et al. (2017) Simulated heat waves during maize reproductive stages alter reproductive growth but have no lasting effect when applied during vegetative stages. Agric. Ecosyst. Environ. 240, 162–170 10.1016/j.agee.2016.11.008

[123] Siebers, M.H., Yendrek, C.R., Drag, D., Locke, A.M., Rios Acosta, L., Leakey, A.D. et al. (2015) Heat waves imposed during early pod development in soybean (*Glycine max*) cause significant yield loss despite a rapid recovery from oxidative stress. Glob. Chang. Biol. 21, 3114–3125 10.1111/gcb.12935 25845935

[124] Lobell, D.B., Bänziger, M., Magorokosho, C. and Vivek, B. (2011) Nonlinear heat effects on African maize as evidenced by historical yield trials. Nat. Clim. Chang. 1, 42–45 10.1038/nclimate1043

[125] Ray, D.K., Gerber, J.S., MacDonald, G.K. and West, P.C. (2015) Climate variation explains a third of global crop yield variability. Nat. Commun. 6, 5989 10.1038/ncomms6989 25609225 PMC4354156

[126] Schlenker, W. and Roberts, M.J. (2009) Nonlinear temperature effects indicate severe damages to US crop yields under climate change. Proc. Natl Acad. Sci. U.S.A. 106, 15594–15598 10.1073/pnas.0906865106 19717432 PMC2747166

[127] Macabuhay, A., Houshmandfar, A., Nuttall, J., Fitzgerald, G.J., Tausz, M. and Tausz-Posch, S. (2018) Can elevated CO_2_ buffer the effects of heat waves on wheat in a dryland cropping system? Environ. Exp. Bot. 155, 578–588 10.1016/j.envexpbot.2018.07.029

[128] Bourgault, M., Löw, M., Tausz-Posch, S., Nuttall, J., Delahunty, A., Brand, J. et al. (2018) Effect of a heat wave on lentil grown under free-air CO_2_ enrichment (FACE) in a semi-arid environment. Crop Sci. 58, 803–812 10.2135/cropsci2017.09.0565

[129] Thomey, M.L., Slattery, R.A., Köhler, I.H., Bernacchi, C.J. and Ort, D.R. (2019) Yield response of field-grown soybean exposed to heat waves under current and elevated [CO_2_]. Glob. Chang. Biol. 25, 4352–4368 10.1111/gcb.14796 31411789

[130] Leakey, A.D., Ainsworth, E.A., Bernacchi, C.J., Rogers, A., Long, S.P. and Ort, D.R. (2009) Elevated CO_2_ effects on plant carbon, nitrogen, and water relations: six important lessons from FACE. J. Exp. Bot. 60, 2859–2876 10.1093/jxb/erp096 19401412

[131] Dai, A., Zhao, T. and Chen, J. (2018) Climate change and drought: a precipitation and evaporation perspective. Curr. Clim. Chang. Rep. 4, 301–312 10.1007/s40641-018-0101-6

[132] Ficklin, D.L. and Novick, K.A. (2017) Historic and projected changes in vapor pressure deficit suggest a continental-scale drying of the United States atmosphere. J. Geophys. Res. Atmos. 122, 2061–2079 10.1002/2016JD025855

[133] Wang, K., Dickinson, R.E. and Liang, S. (2012) Global atmospheric evaporative demand over land from 1973 to 2008. J. Clim. 25, 8353–8361 10.1175/jcli-d-11-00492.1

[134] Yuan, W., Zheng, Y., Piao, S., Ciais, P., Lombardozzi, D., Wang, Y. et al. (2019) Increased atmospheric vapor pressure deficit reduces global vegetation growth. Sci. Adv. 5, eaax1396 10.1126/sciadv.aax1396 31453338 PMC6693914

[135] Hatfield, J.L. and Prueger, J.H. (2015) Temperature extremes: effect on plant growth and development. Weather Clim. Extremes 10, 4–10 10.1016/j.wace.2015.08.001

[136] Zandalinas, S.I. and Mittler, R. (2022) Plant responses to multifactorial stress combination. New Phytol. 234, 1161–1167 10.1111/nph.18087 35278228

[137] Ball, J.T., Woodrow, I.E. and Berry, J.A. (1987) A model predicting stomatal conductance and its contribution to the control of photosynthesis under different environmental conditions. In Progress in Photosynthesis Research: Volume 4 Proceedings of the VIIth International Congress on Photosynthesis Providence, Rhode Island, U.S.A., August 10–15, 1986, pp. 221–224, Springer 10.1007/978-94-017-0519-6

[138] Long, S.P. and Bernacchi, C. (2003) Gas exchange measurements, what can they tell us about the underlying limitations to photosynthesis? Procedures and sources of error. J. Exp. Bot. 54, 2393–2401 10.1093/jxb/erg262 14512377

[139] Lobell, D.B., Hammer, G.L., McLean, G., Messina, C., Roberts, M.J. and Schlenker, W. (2013) The critical role of extreme heat for maize production in the United States. Nat. Clim. Change 3, 497–501 10.1038/nclimate1832

[140] Lobell, D.B., Roberts, M.J., Schlenker, W., Braun, N., Little, B.B., Rejesus, R.M. et al. (2014) Greater sensitivity to drought accompanies maize yield increase in the U.S. Midwest. Science 344, 516–519 10.1126/science.1251423 24786079

[141] Ort, D.R. and Long, S.P. (2014) Limits on yields in the corn belt. Science 344, 484–485 10.1126/science.1253884 24786071

[142] Kupper, P., Sõber, J., Sellin, A., Lõhmus, K., Tullus, A., Räim, O. et al. (2011) An experimental facility for free air humidity manipulation (FAHM) can alter water flux through deciduous tree canopy. Environ. Exp. Bot. 72, 432–438 10.1016/j.envexpbot.2010.09.003

[143] Ort, D.R., Merchant, S.S., Alric, J., Barkan, A., Blankenship, R.E., Bock, R. et al. (2015) Redesigning photosynthesis to sustainably meet global food and bioenergy demand. Proc. Natl Acad. Sci. U.S.A. 112, 8529–8536 10.1073/pnas.1424031112 26124102 PMC4507207

[144] Slattery, R.A. and Ort, D.R. (2019) Carbon assimilation in crops at high temperatures. Plant Cell Environ. 42, 2750–2758 10.1111/pce.13572 31046135

[145] Moore, C.E., Meacham-Hensold, K., Lemonnier, P., Slattery, R.A., Benjamin, C., Bernacchi, C.J. et al. (2021) The effect of increasing temperature on crop photosynthesis: from enzymes to ecosystems. J. Exp. Bot. 72, 2822–2844 10.1093/jxb/erab090 33619527 PMC8023210

[146] Ainsworth, E.A. and Ort, D.R. (2010) How do we improve crop production in a warming world? Plant Physiol. 154, 526–530 10.1104/pp.110.161349 20921178 PMC2949002

[147] Walker, B.J., VanLoocke, A., Bernacchi, C.J. and Ort, D.R. (2016) The costs of photorespiration to food production now and in the future. Annu. Rev. Plant Biol. 67, 107–129 10.1146/annurev-arplant-043015-111709 26865340

[148] Kebeish, R., Niessen, M., Thiruveedhi, K., Bari, R., Hirsch, H.-J., Rosenkranz, R. et al. (2007) Chloroplastic photorespiratory bypass increases photosynthesis and biomass production in *Arabidopsis thaliana*. Nat. Biotechnol. 25, 593–599 10.1038/nbt1299 17435746

[149] Maurino, V.G. and Peterhansel, C. (2010) Photorespiration: current status and approaches for metabolic engineering. Curr. Opin. Plant Biol. 13, 248–255 10.1016/j.pbi.2010.01.006 20185358

[150] South, P.F., Cavanagh, A.P., Liu, H.W. and Ort, D.R. (2019) Synthetic glycolate metabolism pathways stimulate crop growth and productivity in the field. Science 363, aat9077 10.1126/science.aat9077 PMC7745124 30606819

[151] Cavanagh, A.P., South, P.F., Bernacchi, C.J. and Ort, D.R. (2022) Alternative pathway to photorespiration protects growth and productivity at elevated temperatures in a model crop. Plant Biotechnol. J. 20, 711–721 10.1111/pbi.13750 34786804 PMC8989507

[152] Timm, S., Florian, A., Arrivault, S., Stitt, M., Fernie, A.R. and Bauwe, H. (2012) Glycine decarboxylase controls photosynthesis and plant growth. FEBS Lett. 586, 3692–3697 10.1016/j.febslet.2012.08.027 22982108

[153] López-Calcagno, P.E., Fisk, S., Brown, K.L., Bull, S.E., South, P.F. and Raines, C.A. (2019) Overexpressing the H-protein of the glycine cleavage system increases biomass yield in glasshouse and field-grown transgenic tobacco plants. Plant Biotechnol. J. 17, 141–151 10.1111/pbi.12953 29851213 PMC6330538

[154] Bernacchi, C., Calfapietra, C., Davey, P., Wittig, V., Scarascia-Mugnozza, G., Raines, C. et al. (2003) Photosynthesis and stomatal conductance responses of poplars to free-air CO_2_ enrichment (PopFACE) during the first growth cycle and immediately following coppice. New Phytol. 159, 609–621 10.1046/j.1469-8137.2003.00850.x 33873598

[155] June, T., Evans, J.R. and Farquhar, G.D. (2004) A simple new equation for the reversible temperature dependence of photosynthetic electron transport: a study on soybean leaf. Funct. Plant Biol. 31, 275–283 10.1071/FP03250 32688899

[156] Ainsworth, E.A. and Rogers, A. (2007) The response of photosynthesis and stomatal conductance to rising [CO_2_]: mechanisms and environmental interactions. Plant Cell Environ. 30, 258–270 10.1111/j.1365-3040.2007.01641.x 17263773

[157] Zhu, X.-G., De Sturler, E. and Long, S.P. (2007) Optimizing the distribution of resources between enzymes of carbon metabolism can dramatically increase photosynthetic rate: a numerical simulation using an evolutionary algorithm. Plant Physiol. 145, 513–526 10.1104/pp.107.103713 17720759 PMC2048738

[158] Harrison, E.P., Willingham, N.M., Lloyd, J.C. and Raines, C.A. (1997) Reduced sedoheptulose-1, 7-bisphosphatase levels in transgenic tobacco lead to decreased photosynthetic capacity and altered carbohydrate accumulation. Planta 204, 27–36 10.1007/s004250050226

[159] Lefebvre, S., Lawson, T., Fryer, M., Zakhleniuk, O.V., Lloyd, J.C. and Raines, C.A. (2005) Increased sedoheptulose-1, 7-bisphosphatase activity in transgenic tobacco plants stimulates photosynthesis and growth from an early stage in development. Plant Physiol. 138, 451–460 10.1104/pp.104.055046 15863701 PMC1104198

[160] Feng, L., Wang, K., Li, Y., Tan, Y., Kong, J., Li, H. et al. (2007) Overexpression of SBPase enhances photosynthesis against high temperature stress in transgenic rice plants. Plant Cell Rep. 26, 1635–1646 10.1007/s00299-006-0299-y 17458549

[161] Crafts-Brandner, S.J. and Salvucci, M.E. (2000) Rubisco Activase constrains the photosynthetic potential of leaves at high temperature and CO_2_. Proc. Natl Acad. Sci. U.S.A. 97, 13430–13435 10.1073/pnas.230451497 11069297 PMC27241

[162] Kurek, I., Chang, T.K., Bertain, S.M., Madrigal, A., Liu, L., Lassner, M.W. et al. (2007) Enhanced thermostability of Arabidopsis Rubisco activase improves photosynthesis and growth rates under moderate heat stress. Plant Cell 19, 3230–3241 10.1105/tpc.107.054171 17933901 PMC2174701

[163] Scafaro, A.P., Bautsoens, N., den Boer, B., Van Rie, J. and Gallé, A. (2019) A conserved sequence from heat-adapted species improves Rubisco activase thermostability in wheat. Plant Physiol. 181, 43–54 10.1104/pp.19.00425 31189658 PMC6716234

[164] Scafaro, A.P., De Vleesschauwer, D., Bautsoens, N., Hannah, M.A., den Boer, B., Gallé, A. et al. (2019) A single point mutation in the C-terminal extension of wheat Rubisco activase dramatically reduces ADP inhibition via enhanced ATP binding affinity. J. Biol. Chem. 294, 17931–17940 10.1074/jbc.RA119.010684 31530638 PMC6879333

[165] Degen, G.E., Worrall, D. and Carmo-Silva, E. (2020) An isoleucine residue acts as a thermal and regulatory switch in wheat Rubisco activase. Plant J. 103, 742–751 10.1111/tpj.14766 32363739

[166] Kumagai, E., Burroughs, C.H., Pederson, T.L., Montes, C.M., Peng, B., Kimm, H. et al. (2022) Predicting biochemical acclimation of leaf photosynthesis in soybean under in-field canopy warming using hyperspectral reflectance. Plant Cell Environ. 45, 80–94 10.1111/pce.14204 34664281

[167] Kimm, H., Guan, K., Burroughs, C.H., Peng, B., Ainsworth, E.A., Bernacchi, C.J. et al. (2021) Quantifying high-temperature stress on soybean canopy photosynthesis: the unique role of sun-induced chlorophyll fluorescence. Glob. Chang. Biol. 27, 2403–2415 10.1111/gcb.15603 33844873

